# Imaging of the Pancreas in New-Onset Diabetes: A Prospective Pilot Study

**DOI:** 10.14309/ctg.0000000000000478

**Published:** 2022-03-25

**Authors:** Bechien U. Wu, Eva Lustigova, Qiaoling Chen, Elizabeth Y. Dong, Anirban Maitra, Suresh T. Chari, Ziding Feng, Jo Ann Rinaudo, Lynn M. Matrisian, Rex A. Parker

**Affiliations:** 1 Center for Pancreatic Care, Kaiser Permanente Los Angeles Medical Center, Los Angeles, California, USA;; 2 Department of Research and Evaluation, Kaiser Permanente Southern California, Pasadena, California, USA;; 3 University of Texas, MD Anderson Cancer Center, Houston, Texas, USA;; 4 Fred Hutchinson Cancer Center, Seattle, Washington, USA;; 5 National Cancer Institute, Division of Cancer Prevention, National Cancer Institute, Bethesda, MD, USA;; 6 Pancreatic Cancer Action Network, Manhattan Beach, California, USA;; 7 Department of Radiology, Kaiser Permanente Los Angeles Medical Center, Los Angeles, California, USA.

## Abstract

The aim of this study was to assess the feasibility of cross-sectional imaging for detection of pancreatic cancer (PDAC) in patients with new-onset hyperglycemia and diabetes (NOD).

**METHODS:**

We conducted a prospective pilot study from November 2018 to March 2020 within an integrated health system. Patients aged 50–85 years with newly elevated glycemic parameters without a history of diabetes were invited to complete a 3-phase contrast-enhanced computed tomography pancreas protocol scan while participating in the Prospective Study to Establish a NOD Cohort. Abnormal pancreatic findings, incidental extrapancreatic findings, and subsequent clinical evaluation were identified. Variability in clinical reporting between medical centers based on descriptors of pancreatic duct and parenchyma was assessed.

**RESULTS:**

A total of 130 of 147 participants (88.4%) consented to imaging; 93 scans were completed (before COVID-19 stay-at-home order). The median age was 62.4 years (interquartile range 56.3–68.8), 37.6% women; Hispanic (39.8%), White (29.0%), Black (14.0%), and Asian (13.3%). One (1.1%) case of PDAC (stage IV) was diagnosed, 12 of 93 participants (12.9%) had additional pancreatic findings: 5 fatty infiltration, 3 cysts, 2 atrophy, 1 divisum, and 1 calcification. There were 57 extrapancreatic findings among 52 of 93 (56%) unique patients; 12 of 57 (21.1%) prompted clinical evaluation with 2 additional malignancies diagnosed (nonsmall cell lung and renal oncocytoma). Reports from 1 participating medical center more frequently provided description of pancreatic parenchyma and ducts (92.9% vs 18.4%), *P* < 0.0001.

**DISCUSSION:**

High proportion of incidental findings and variability in clinical reports are challenges to be addressed for a successful NOD-based early detection strategy for PDAC.

## INTRODUCTION

Pancreatic cancer (PDAC) is the 11th most common cancer in the United States but is the third leading cause of cancer-related death with an estimated 47,050 deaths in 2020 (1). Contributing to the lethal nature of this cancer is the fact that many patients (52%) are diagnosed at a late stage of disease with distant metastases (1). This is in large part due to the lack of symptoms during early stages of PDAC. However, because of the relative rarity of this cancer, widespread population-based screening is unlikely to be effective and is not currently recommended by the United States Preventive Services Task Force (2). Nevertheless, the ability to identify patients with tumor before the onset of overt symptoms offers an opportunity to affect the natural history of this disease, leading to improved outcomes as patients diagnosed with local, resectable disease have improved overall survival (1). A targeted approach to screening among patients at increased risk for PDAC based on familial or genetic risk factors offers a potential early detection strategy (3) but represents only 5%–10% of overall cases of PDAC.

New-onset diabetes (NOD) among persons aged 50 or older has been identified as a potential early indicator of PDAC with a 6–8-fold risk compared with the general population and has been described in up to 25% of cases of PDAC (4–8). To further explore the relationship between diabetes and PDAC, the Prospective Study to Establish a New-Onset Hyperglycemia and Diabetes (NOD) Cohort was initiated in 2018 (9). The objective of this multicenter prospective cohort study is to validate previous retrospective findings related to the incidence of PDAC, establish a biospecimen repository for future biomarker testing, and develop a platform for future targeted screening studies in PDAC. The aim of this study was to inform the development of such a targeted screening strategy.

Multidetector computed tomography (MDCT) using a pancreatic protocol is currently the most widely used imaging study for the initial diagnosis and staging of PDAC (10,11). However, overall experience with MDCT for early detection of PDAC is limited. We therefore sought to conduct a pilot study to assess the feasibility of MDCT-based screening for PDAC among patients with new-onset hyperglycemia and/or diabetes at the time of elevated qualifying glycemic parameter. Specifically, we aimed to assess participant acceptance of the image-based screening protocol, frequency of pancreatic as well as extrapancreatic findings, and variability in reporting of imaging findings.

## METHODS

### Protocol development

An in-person planning meeting was convened on July 9, 2018, on the campus of the National Cancer Institute in Shady Grove, Maryland. A multidisciplinary panel of experts were invited to participate in protocol development for the Early Detection Initiative in Pancreatic Cancer, including a breakout session devoted specifically to imaging tests to be performed as part of a potential screening study for PDAC among patients with NOD. The panel discussed various options for imaging-based screening, including MDCT, MRI, and endoscopic ultrasound (EUS). A detailed synopsis of the options presented and the rationale provided for each strategy is presented in the Supplementary Digital Content (see Supplementary Material, http://links.lww.com/CTG/A785). Ultimately, MDCT was selected as the test of choice given concerns over the availability and costs of some of the other testing modalities. In addition, the panel indicated potentially reduced interobserver variability in interpretation of computed tomography (CT) imaging compared with MRI or EUS.

### Study design and setting

This prospective pilot study was conducted from November 2018 to April 2020 as an ancillary study of the Consortium for the Study of Chronic Pancreatitis, Diabetes, and Pancreatic Cancer (National Institute of Diabetes, Digestive and Kidney diseases, and National Cancer Institute) and Early Detection Initiative (Pancreatic Cancer Action Network). The study was approved by the Institutional Review Board of Kaiser Permanente Southern California (KPSC). All participants enrolled in the NOD Cohort at the KPSC site were offered optional research-related MDCT during the pilot study period to better characterize potential implications of imaging-based screening in this patient population. KPSC is a regional integrated health system that provides comprehensive healthcare services for over 4 million members. Informed consent was obtained from all study participants before completion of any research procedures.

### Study population

Patients aged 50–85 years with newly elevated glycemic parameters (elevated fasting glucose ≥126 mg/dL, random glucose ≥200 mg/dL, 2-hour oral glucose tolerance test ≥200 mg/dL with subsequent confirmatory test, or single glycated hemoglobin ≥6.5%) without a history of diabetes or previous elevation in glycated hemoglobin were eligible for study participation. In addition, patients who were initiated on antihyperglycemic medication after a newly elevated glycemic parameter were considered eligible for study participation. All participants were also required to have evidence of nonelevated glycemic parameters within 18 months before to verify new-onset status of hyperglycemia. Patients with a history of pancreatic cancer or undergoing treatment for an alternative malignancy were excluded from study participation. Additional aspects of the study inclusion and exclusion criteria have been published previously (9). In addition, patients unwilling or unable to undergo contrast-enhanced computed tomography were excluded from study participation.

### Study procedures

#### Participant identification and recruitment

Patients who fulfilled eligibility criteria and enrolled in the NOD Cohort were invited to participate in the current pilot study to assess feasibility of MDCT-based screening for PDAC.

#### Imaging protocol and interpretation

All MDCT Pancreatic Adenocarcinoma Protocol were performed per the consensus statement of the Society of Abdominal Radiology and the American Pancreatic Association and as indicated in National Comprehensive Cancer Network Guidelines version 2.2019 (10,11). Initial interpretation of all imaging studies was performed by practicing clinical radiologists within KPSC, and findings were entered into the patient's medical record in accordance with standard clinical practice. Study physicians were responsible for communicating any potentially clinically relevant findings with the participant's primary care team. In addition to the clinical review, a dedicated research reading was performed by a study radiologist blinded to the original clinical interpretation.

#### Clinical outcome assessment

Participant's electronic health records were manually reviewed to assess subsequent diagnostic testing performed as a result of imaging findings performed in the context of this study. Attribution of further testing to image-based findings from the research study was confirmed based on physician clinical documentation referencing the imaging findings as indication for subsequent evaluation. Clinical outcomes were assessed up to 6 months after completion of study-related imaging.

### Data analysis

#### Feasibility

As a pilot study we aimed to assess feasibility of recruitment with a target sample size of n = 100 participants. Feasibility objectives included accrual rate and completion rate of MDCT. We further sought to evaluate racial/ethnic representation within the initial study cohort to identify any potential bias in study recruitment or outreach efforts.

#### Imaging analysis

We evaluated several aspects of MDCT-based imaging among NOD study participants. In addition to the timing of image completion, we assessed variability with respect to clinical reporting of pancreatic imaging findings across the 3 KPSC imaging centers. We developed a numeric scoring system to objectively measure the extent of reporting wherein 1 point was assigned for each mention regarding the presence or absence of either parenchymal or ductal abnormalities of the pancreas. In addition, an independent secondary review was completed by a dedicated study radiologist who was blinded to the clinical report findings. The research interpretation of images included assessment for a series of findings categorized *a priori* as suspicious for PDAC (mass, stricture, or main duct dilatation), as well as additional pancreatic findings (cysts, pancreatitis, atrophy, calcification(s), fatty pancreas, gallstones, intrapancreatic biliary duct dilatation/stone, and intrahepatic biliary duct dilatation). Extrapancreatic findings potentially related to PDAC were further assessed including liver lesions, omental nodules or ascites, and lymphadenopathy. Extrapancreatic findings unrelated to PDAC were classified according to the following:

1. E1: normal examination or normal variant.
2. E2: clinically unimportant finding, e.g., simple liver cyst and vertebral hemangioma: no workup required.
3. E3: likely unimportant or incompletely characterized finding, e.g. minimally complex renal cyst: referral depends on local center and standards of care.
4. E4: potentially important finding, e.g., solid renal mass and abdominal aortic aneurysm: communicate to referring physician.

#### Statistical analysis

The distributions of baseline demographic and clinical characteristics of the study cohort were presented using mean (SD), median (interquartile range [IQR]), or number of patients (percentage) as appropriate. For overlapping features noted in both clinical and research assessments, we calculated the joint probability of agreement. This was estimated as the proportion of clinical and research review agreement with respect to individual findings further assessed by Cohen Kappa and its 95% confidence interval.

## RESULTS

**Figure 1.**
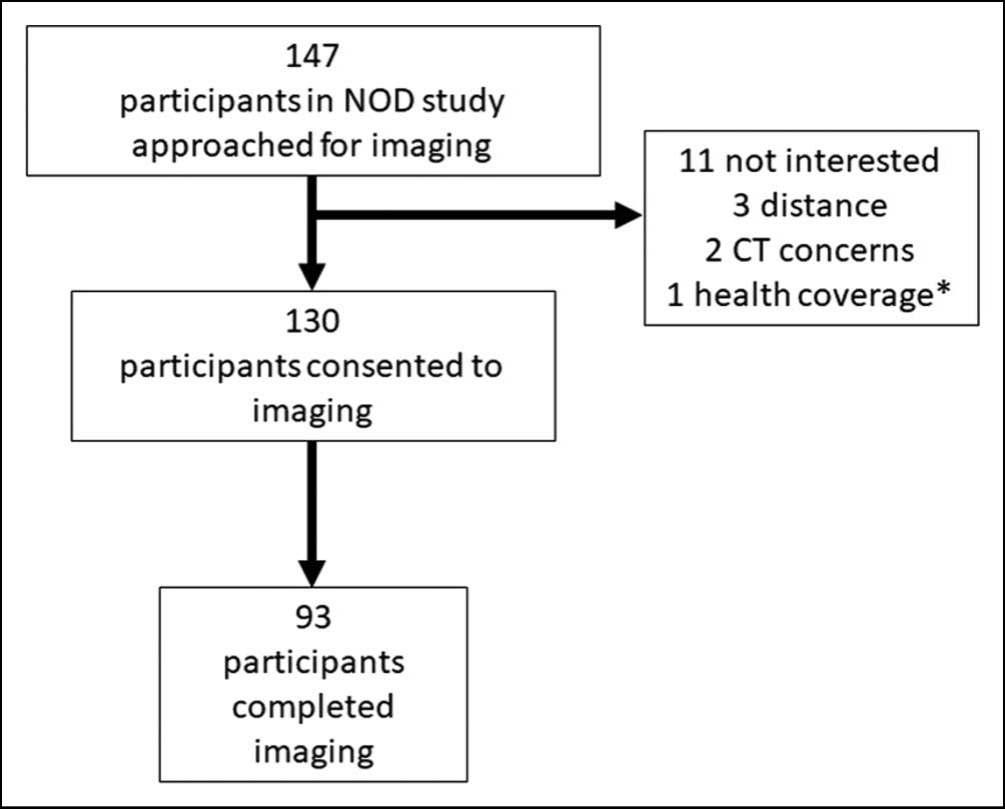
Participant enrollment flowdiagram. *Individual was no longer a KPSC health plan member at time of recruitment and opted out of the imaging protocol. CT, computed tomography; NOD, new-onset hyperglycemia and diabetes.

During the study's 17-month accrual period, a total of 147 participants were enrolled in the NOD Cohort at the KPSC site. Among the 147 enrolled study participants, 130 (88.7%) consented to undergo MDCT. A total of 93 patients completed MDCT before initiation of the California state COVID-19 stay-at-home order in March 2020 for an average accrual rate of 5.4 participants/month. A flowdiagram for cohort assembly is presented in Figure 1.

The median age was 62.4 years (IQR 56.3–68.8), and 37.8% were women. The racial/ethnic composition of the study cohort was broadly representative of the KPSC membership: Hispanic (39.8%), White (29.0%), Black (14.0%), and Asian (12.9%). Baseline demographic and clinical characteristics of the study cohort are presented in Table 1.

**Table 1.** Patient demographic and clinical characteristics, N = 93

Age median (Q1–Q3)	62.4 (56.3–68.8)
Race/ethnicity, n (%)	
White	27 (29%)
Black	13 (14%)
Hispanic	37 (39.8%)
Asian/Pacific Islander	12 (12.9%)
Other/unknown	4 (4.3%)
Sex	
Female	35 (37.6%)
Male	58 (62.4%)
Length of membership (yr), median (Q1–Q3)	14.7 (4.9–31.2)
Body mass index, mean (SD)	34.0 (6.22)
Normal weight	4 (4.3%)
Overweight	21 (22.6%)
Obese	67 (72%)
Unknown	1 (1.1%)
Alcohol	
No	39 (41.9%)
Yes	48 (51.6%)
Unknown	6 (6.5%)
Smoking	
Nonsmoker	64 (68.8%)
Quit smoking	23 (24.7%)
Current smoker	3 (3.2%)
Unknown	3 (3.2%)
Charlson comorbidities, n (%)	
0	25 (26.9%)
1	47 (50.5%)
2+	21 (22.6%)
NOD eligibility	
Elevated A1c with antidiabetes medication	16 (17.2%)
Elevated A1c with consecutive a1c/glucose	18 (19.4%)
Elevated A1c with simultaneous glucose	23 (24.7%)
Single elevated A1c	36 (38.7%)
Index PDM (A1c)	
Mean (SD)	7.3 (1.49)
Median (Q1–Q3)	6.7 (6.5–7.1)
Range	6.5–14.3
Days from order to CT scan, median (Q1–Q3)	19 (15–27.5)
Days from index PDM to CT scan, median (Q1–Q3)	93 (64–116.5)
Distance from home to where CT was taken(mile), median (Q1–Q3)	10.7 (6.3–20.4)

CT, computed tomography; NOD, new-onset hyperglycemia and diabetes.

The median time from order requisition to completion of imaging was 19 days (IQR 15–27.5). The median time from qualifying laboratory parameter to completion of imaging was 93 days (IQR 64–116.5). There were no immediate adverse events noted related to MDCT imaging. There was 1 (1.1%) case of PDAC diagnosed among enrolled study participants. This patient developed jaundice several weeks after initial enrollment, prompting them to seek medical attention. Subsequent imaging demonstrated a 4-centimeter mass in the head of the pancreas with likely hepatic metastasis that was subsequently confirmed on biopsy of the liver lesion. In addition, 12 of 93 (13%) patients had pancreatic findings noted during a clinical review of their MDCT scans: 5 fatty infiltration, 3 pancreatic cysts (size <5, 9 mm and 2.3 cm), 2 atrophy, 1 pancreas divisum, and 1 calcification. Among these, 1 of 12 (8.3%) participants underwent further diagnostic evaluation (endosonography for further evaluation of 2.3 cm pancreatic cystic neoplasm: fine-needle aspiration was not performed). All patients with pancreatic cysts have been scheduled to undergo further routine surveillance in accordance with KPSC regional pancreatic cyst surveillance recommendations adapted from Gastroenterology and Radiology society guidelines (12–15). There were 57 clinically relevant extrapancreatic findings among 52 of 93 (56%) unique patients, 12 of 57 (21.1%) prompted clinical evaluation (E4) leading to diagnosis of 2 extrapancreatic malignancies (stage IV multifocal lung adenocarcinoma, localized renal oncocytoma). A summary of both the pancreatic and extrapancreatic findings that prompted subsequent testing is presented in Table 2. Reports from 1 participating medical center (tertiary referral center) more frequently provided description of pancreatic parenchyma and ducts (92.9% vs 18.4%), *P* < 0.0001.

**Table 2.** Pancreatic and extrapancreatic findings prompting further clinical evaluation, n = 30

	Diagnostic testing	Clinical outcome
Pancreatic cancer (1)	1 liver biopsy	Treatment
Pancreatic lesions (3 cystic lesions)	1 MRI 1 EUS	Staged surveillance program
Liver lesions (8)	3 MRI 1 abdominal ultrasound	3 hemangiomas Normal
Pulmonary nodule (6)	1 percutaneous biopsy and subsequent wedge resection	1 lung adenocarcinoma
Adrenal lesion (6)	1 CT adrenal protocol	1 adrenal nodule
Renal lesion (3)	1 CT renal followed by partial nephrectomy	1 renal oncocytoma
Genitourinary tract lesion (3)	1 transvaginal ultrasound 1 pap smear 1 serum PSA	Uterine fibroids Normal Normal

CT, computed tomography; EUS, endoscopic ultrasound; MRI, magnetic resonance imaging; PSA, prostate-specific antigen.

Based on the independent research review, there were an additional 17 cases with pancreatic parenchymal atrophy and 1 instance of main pancreatic duct dilatation identified that were not indicated on the clinical reports of the CT images. Agreements between the clinical reports and research review (where applicable) are presented in Table 3.

**Table 3.** Interrater agreements, clinical vs research image review

	Clinical reading	Research reading	% Agreement	Kappa (95% confidence interval)
Atrophy	3 (3.2%)	19 (20.4%)	82.80%	0.23 (0.01–0.45)
Calcification	1 (1.1%)	0 (0%)	98.90%	NA
Cyst	3 (3.2%)	2 (2.2%)	98.90%	0.79 (0.40–1.00)

## DISCUSSION

In this prospective pilot study, we demonstrated feasibility of timely accrual of participants with new-onset hyperglycemia based on an automated electronic search algorithm. In addition, enrolled participants were of diverse racial/ethnic background and for the most part were willing to complete pancreas protocol computed tomography as a component of their study participation. Three participants (3.3%) had clinically significant findings identified with respect to the pancreas including 1 PDAC diagnosed during the study period. Two additional extrapancreatic tumors were diagnosed as a result of study participation.

Newly diagnosed (incident) diabetes in persons after age 50 has received attention as a potential marker of early or undiagnosed PDAC with 3-year cancer rates ranging from 0.25% to 1.0% (4–7,16,17). Notably studies that have reported the highest incidence incorporated glycemic criteria (5–7) rather than physician diagnosis codes (16). This distinction may be related to potential delays in clinical diagnosis or inaccurate coding. In addition, our previous work has demonstrated an increased risk of PDAC in the setting of new-onset hyperglycemia based on glycated hemoglobin levels with 3-year incidence of 0.77 per 1,000 person-years with the highest risk of PDAC within the first year after new-onset hyperglycemia (18). Therefore, timely identification, recruitment, and image acquisition of participants soon after the development of hyperglycemia is a key component to achieving the aims of the prospective NOD cohort study and any potential early detection strategy.

Several attempts have been made to translate the observed relationship between NOD and PDAC into a potential strategy for early detection. A study based on endoscopic retrograde pancreatography from Japan identified 5 (13.5%) cases of advanced stage PDAC among 36 participants with recently (<3 months) diagnosed diabetes (19). A subsequent study of 115 participants from Hungary applied CA 19-9 and transabdominal ultrasound in patients within 3 months of newly diagnosed diabetes (20). A total of 3 subjects (2.6%) were identified with PDAC, all of whom were inoperable. Limitations of these previous approaches include the invasive nature of endoscopic retrograde pancreatography and lack adequate sensitivity of CA 19-9 that has been reported a sensitivity of 78% (range 70%–90%). In addition, it is unclear whether transabdominal ultrasound has sufficient resolution for detection of early pancreatic tumors, especially compared with cross-sectional imaging. In the Cancer of the Pancreas Screening cohort (a longitudinal study of 351 high-risk individuals based on either genetic or family history) 10 of 11 screen-detected cancers were resectable (3,21). The CAPS study used a combination of magnetic resonance imaging and EUS for identification of pancreatic lesions. Although such an intensive screening protocol may be justified and even cost-effective (22–24) in specific high-risk populations whose risk of PDAC can approach 30 times the general population, it is unlikely to be feasible in patients with NOD whose risk is estimated to be 6–8-fold compared with the general population (25).

Additional approaches for further risk stratification have been proposed to identify a subset of patients with NOD at increased risk for PDAC. These include clinical prediction models such as a regression-based model derived from the United Kingdom Health Improvement Network (26) and the 3 variable Enriching NOD model for PDAC (END-PAC) derived on data from the Rochester Epidemiology Project (27). The END-PAC model has since been validated in 2 independent retrospective cohorts with an area under curve of 0.72–0.75 (28,29) and is the basis for an active clinical trial on early detection of PDAC (NCT04662879). If successful, data support this approach as a potentially cost-effective strategy for early detection of PDAC among patients with NOD (30).

Imaging is a key component for any successful early detection strategy in PDAC. We therefore sought to assess the potential role of MDCT for detection of pancreatic lesions in this study population. This study demonstrated the feasibility of rapid patient identification based on real-time laboratory results and completion of imaging within 120 days from qualifying laboratory test results. The most noted pancreas-related finding, fatty pancreas, was identified in 5% of study participants. Although the implications of fatty pancreas are not yet well established, this condition has been increasingly recognized as a component of the metabolic syndrome (31) and has potential to be related to development of chronic pancreatitis as well as PDAC. Two additional malignancies were identified as a result of imaging obtained in the context of this study, indicating potential opportunities for early detection of cancers beyond the pancreas in the NOD population.

Findings from this study also highlight concerns associated with an MDCT-based strategy for early detection of PDAC. In addition to the 3 patients with clinically significant pancreatic findings, there was a substantial proportion (>50%) with incidental extrapancreatic findings. Twelve of the study participants (13%) underwent further clinical evaluation based on their imaging findings. As several of these cases were associated with preclinical detection of extrapancreatic tumors, it is arguable whether these clinical evaluations represent a harm or net benefit associated with screening. Nevertheless, the likelihood of downstream extrapancreatic evaluations based on incidental findings should be factored into the costs associated with any strategy that uses cross-sectional imaging for early detection of PDAC.

There are additional challenges for early detection of PDAC based on new-onset hyperglycemia. Despite real-time patient identification based on laboratory test results and timely enrollment of eligible participants, the single case of PDAC study was already at an advanced stage. Although previous studies have indicated NOD (6,7,32) and elevation in glycated hemoglobin (18) can occur as early 36 months before the diagnosis of PDAC, it remains to be determined whether imaging performed at the time of hyperglycemia can lead to detection of PDAC at an early, potentially curable stage. Although patients were able to complete imaging within a median of 93 days from laboratory abnormality, it is unclear whether this is an adequate time frame to affect the overall disease course with respect to PDAC. We also noted significant variability in the reporting of pancreatic parenchymal and ductal features in clinical reports of the CT scans performed. Standardization of reporting of potentially relevant findings is another key step for successful early detection (10). In this study, descriptors of the pancreas and duct were more frequently provided from the tertiary care center potentially related to availability of subspecialty focused radiologists. An example of such a finding that was noted on the research review but absent from the clinical reporting is dilatation of the main pancreatic duct. Duct dilatation is a particularly relevant imaging finding as previous retrospective studies have indicated that abnormalities of the pancreatic duct can be detected on cross-sectional imaging in 50% of cases up to 18 months before a diagnosis of PDAC (33). Among patients with main pancreatic duct dilatation noted on cross-sectional imaging, 10% were ultimately diagnosed with PDAC within 3 years in another retrospective cohort study (34).

Strengths of this study include the diverse racial/ethnic composition of the study cohort, ability to leverage real-time laboratory data for rapid participant accrual, and the integrated care setting that facilitated timely communication of potentially significant findings with a patient's primary care team. Limitations included an interruption of accrual in March 2020 due to the COVID-19 pandemic. Unfortunately, this was an unforeseen circumstance that could not be overcome and affected nearly all clinical research during this period. However, beyond this interruption, it is also likely that the participation rate noted in this study (88.7%) exceeds what would be observed in the setting of a clinical trial or real-world setting. This is largely due to a recruitment strategy that relied on approaching patients who had already agreed to participate in the NOD Cohort. An additional limitation was the inability to accurately assess correlation between clinical reporting compared with research review due to lack of descriptors provided in many of the clinical reports. The increased number of extrapancreatic tumors identified compared with PDAC in this study also indicates the lack of specificity associated with CT-based screening as an approach to early detection.

In conclusion, an imaging-based screening protocol that incorporates multidetector pancreas protocol computed tomography and patient outreach based on real-time laboratory results was feasible in patients with NOD. Although this study sample size was insufficient to draw conclusions related to the potential effectiveness of this approach, our findings highlight several remaining challenges for early detection of PDAC in this patient population. These include a high frequency of incidental extrapancreatic findings, variability in clinical reporting of images, and a late stage of PDAC that was diagnosed. These considerations will need to be addressed to successfully translate the observed relationship between NOD and PDAC into an effective strategy for early detection.

## CONFLICTS OF INTEREST

**Guarantor of the article:** Bechien U. Wu, MD.

**Specific author contributions:** Study concept: B.W. and R.P. Study design: B.W., Z.F., A.M., S.C., J.R., and L.M. Data acquisition and analysis: R.P., E.L., Q.C., and E.D. Interpretation of data: B.W., Z.F., A.M., S.C., J.R., and L.M. Drafting of manuscript: B.W., E.D., and E.L. Critical review of manuscript: A.M., Z.F., S.C., L.M., J.R., E.D., Q.C., E.L., and R.P.

**Financial support:** Research in this publication was supported by the National Cancer Institute and National Institute of Diabetes and Digestive and Kidney Diseases of the National Institutes of Health under award number(s) related to The Consortium for the Study of Chronic Pancreatitis, Diabetes, and Pancreatic Cancer (CPDPC), U01DK108328, U01DK108288, U01DK108314 and the Pancreatic Cancer Action Network.

**Potential competing interests:** A.M. receives royalties for a pancreatic cancer biomarker test from Cosmos Wisdom Biotechnology, and this financial relationship is managed and monitored by the UTMDACC Conflicts of Interest Committee. A.M. also receives royalties as an inventor on a patent that has been licensed by Johns Hopkins University to Thrive Earlier Detection (an Exact Sciences Company). All remaining authors affirm no potential conflicts to disclose.

**Disclaimer:** The content is solely the responsibility of the authors and does not necessarily represent the official views of the National Institutes of Health.

**Study Highlights**

**WHAT IS KNOWN**

✓ Currently, there are no established strategies for early detection of sporadic pancreatic cancer (PDAC).
✓ New-onset diabetes after age 50 may be an indicator of PDAC.

**WHAT IS NEW HERE**

✓ We identified a case of PDAC before clinical diagnosis through use of an automated electronic algorithm based on evaluation of glycemic parameters.
✓ The high proportion of incidental findings and late stage of PDAC at diagnosis may limit an imaging-based strategy for early detection of PDAC based on new-onset diabetes.
✓ Variability in reporting suggests the need for uniform standards for interpretation of high-risk features for PDAC.

## Supplementary Material
