# Supplementary material for: Imaging of the Pancreas in New-Onset Diabetes: A Prospective Pilot Study
Source: Clin Transl Gastroenterol. 2022 Mar 25;13(6):e00478. doi: 10.14309/ctg.0000000000000478 (PMC9236602; doi:10.14309/ctg.0000000000000478)
Supplement: Supplementary file 1 [file ct9-13-e00478-s001.pdf]

IMAGING WORKING GROUP RECOMMENDATIONS

July 9, 2018 Early Detection Initiative meeting

Overall goal: show a stage-shift in diagnosis of pancreatic ductal adenocarcinoma (PDAC)
- Detect Stage 1 tumor (<2cm) at 75% sensitivity

Working Group charge: Guidance for Early Detection Initiative (EDI) protocol on:
- Imaging modality (CT vs MRI)
  - Specific instrument parameters
- Criteria for repeat imaging (baseline/serial)
- Clinical workup (EUS, etc)
- Follow-up on ambiguous findings in the pancreas
- Follow-up on ambiguous findings outside the pancreas

Imaging modality: Various modalities were discussed with pro's and con's relevant to the EDI study design. Research CT was identified as the most favorable initial imaging approach, followed by clinical standard-of-care, which in most cases will be by endoscopic ultrasound (EUS). MRI can be performed if CT contrast is contraindicated.

Imaging approach	Pro	Con	Relative Rank
Research CT, if positive clinical EUS	Easiest to obtain<br>Least expensive	Negative CT may miss lesions	High
Research MRI, if positive, clinical EUS	No radiation<br>Superior resolution<br>More heterogeneity<br>More pancreatic incidental findings	Cost (with equivalent sensitivity)<br>Lesser availability<br>More heterogeneity<br>More pancreatic incidental findings	Low
Research CT & Research EUS	Most exhaustive sensitivity<br>More diagnostic yield<br>Enables comparison	Greater risk<br>Greater cost<br>Potential accrual problem	High
Research CT & Research MRI	Potentially higher sensitivity<br>Non-invasive<br>Enables comparison	May still need clinical EUS<br>More incidental findings	Low
Research CT & Research MRI & Research EUS	Highest sensitivity<br>Highest diagnostic performance	Highest cost<br>Potential accrual problem	Medium

Instrument parameters: Multidetector CT
Slice: 8 slice scanner as minimum
Imaging phases:

Pre-contrast phase (optional)
Pancreatic protocol phase
PV phase (required)

Delayed phase (optional)
IV contrast: 3-5 cc (with 5 cc preferred)
Slice thickness: 2.5 mm (minimum), 1 mm (preferred)
Coronal and sagittal reformations

Repeat imaging: at 6 months if first is negative

Ambiguous findings in pancreas:
Cysts
Calcifications
Other masses
Main duct dilation
Fatt pancreas
Pancreatitis
atrophy

Ambiguous findings outside pancreas:
ERADS E0-E4 grading
Standard of care follow-up

EARLY DETECTION INITIATIVE
For Pancreatic Cancer

NCI/PanCAN Early Detection Initiative for Pancreatic Cancer

Kick-Off Meeting Agenda

July 9, 2018

NCI Shady Grove Campus | 2E 908

8:30am – 8:45am	Welcome from Steering Committee	*Lowy, Matrisian and Chari*
8:45am – 9:45am	Overview of New Onset Diabetes cohort study and vision for EDI	*Suresh Chari*
9:45am – 10:00am	Charge to the Committees	*Lynn Matrisian*
10:00am – 10:30am	Break | Discussion	
10:30am – 12:30pm	Breakout Sessions around specific topics	*All participants*
12:30pm - 1:00pm	Lunch (on your own)	
1:00pm – 2:00pm	Additional break-out time around topics not covered	
2:00pm – 2:30pm	Committee Chairs Meet | Networking for Members	
2:30pm – 4:30pm	Report-out of Committees – 20 mins each (10 min presentation, 10 min discussion)	

Design Committee .... *Ziding Feng*

Imaging WG .... *Walter Park*

Biomarkers .... *Anirban Maitra*

Modeling WG .... *Bechien Wu*

Recruitment and Retention Committee .... *Barbara Kenner*

4:30pm – 5:00pm	Wrap-up and Closing Remarks	*Steering Committee*

Committees and Break-Out Topics:

Members	Meeting Objectives/Topics
**Steering Committee**	
Douglas Lowy, Deputy Director, NCI<br>Suresh Chari, Project PI, Mayo Clinic<br>Lynn Matrisian, CSO, PanCAN	
**Design Committee | Room 2E 034**	
**Ziding Feng, MDA, Chair**<br>Steven Piantadosi, Harvard U<br>Philip Prorok, NCI<br>Yingqu Zhao, Fred Hutchinson<br>William Hoos, PanCAN support	• Overall biostatistical design<br>○ For model validation/use<br>○ For biomarker validation<br>○ For stage shift
**Sieves Committee/Imaging WG | Room 2E 904**	
**Walter Park, Stanford, Chair**<br>Eric Tamm, MDA<br>Avinash Kambadakone, MGH<br>Eugene Koay, MDA<br>Elliot Fishman, Johns Hopkins<br>David Weinberg, Fox Chase, ACRIN<br>Guillermo Marquez, NCI<br>Cassadie Moravek, PanCAN support	• Elements of EDI protocol<br>○ CT vs MRI, protocol<br>○ Triggers for clinical work-up and repeat imaging<br>○ Ambiguous finding in pancreas<br>○ Incidental findings in and outside of the pancreas<br>• How to detect a <2cm tumor for stage shift
**Sieves Committee/Biomarkers WG | Room 2E 032**	
**Anirban Maitra, MDA, Chair of WG and Sieves Committee**<br>Rosalie Sears, OHSU<br>Brian Wolpin, Harvard U<br>Eithne Costello-Goldring, Liverpool<br>Mike Goggins, Johns Hopkins<br>Sudhir Srivastava, NCI<br>Maya Bader, PanCAN support	• Criteria for use of retrospective samples<br>• Parameters of success for a validated biomarker<br>• Criteria for eventual incorporation into a prospective study<br>• Criteria for incorporation of additional sample collection

<table>
<tr><th>Members</th><th>Meeting Objectives/Topics</th></tr>
<tr><td colspan="2">Sieves Committee/Modeling WG | Room 2W 904</td></tr>
<tr><td>Bechien Wu, KPSC, Chair
Yu-Xiao Yang, U Penn
Stephen van den Eeden, KPNC
Ayush Sharma, Mayo Clinic
Kevin Dodd, NCI
Amy Westermann, PanCAN support</td><td>• Criteria to determine if a model is ready to be incorporated up-front into study
• Elements of EDI protocol
○ Follow up of H, M, L risk groups
○ Modeling beyond baseline – reapplying at different time points
○ Specimen collection for low risk group</td></tr>
<tr><td colspan="2">Recruiting and Retention Committee | Room 2E 908</td></tr>
<tr><td>Barbara Kenner, KFRF, Chair
Eva Lustigova, KPSC
Allison McElvaine, ADA
Richard Frank, Western Conn
Gwendolyn Parker, VCU (NCORP)
Worta McCaskill-Stevens, NCI
Kieran Mather, IU
Ashok Saluja, U Miami
Donna Manross, PanCAN Support</td><td>• Develop a strategic plan that will clarify and manage barriers of recruitment for each site and facilitate timely recruitment
○ Process for contacting individuals identified by eligibility algorithm
○ Process for contacting individuals identified by clinical model
○ Barriers and potential solutions to recruiting – physician & staff barriers
○ Recruitment enhancements</td></tr>
<tr><td colspan="2">Core Team</td></tr>
<tr><td>Lola Rahib, PanCAN
Jo Ann Rinaudo, DCP, NCI
Jackie Dahlgren, Fred Hutchinson
Valerie Holzherr, PanCAN Support
Emily Lyons, PanCAN operations Support</td><td>Suresh Chari (see above)
Anirban Maitra (see above)
Lynn Matrisian (see above)
Barbara Kenner (see above)
Sudhir Srivastava (see above)
William Hoos (see above)</td></tr>
<tr><td colspan="2">Special Guests</td></tr>
<tr><td>Stephen James, NIDDK
Steve Pandol, Cedars-Sinai
Bernard Parker, NCI, NCORP
Robert Martin, U Louisville</td><td></td></tr>
</table>

GREY – unable to attend July 9th meeting
